# Generating Functional and Highly Proliferative Melanocytes Derived from Human Pluripotent Stem Cells: A Promising Tool for Biotherapeutic Approaches to Treat Skin Pigmentation Disorders

**DOI:** 10.3390/ijms24076398

**Published:** 2023-03-29

**Authors:** Manoubia Saidani, Annabelle Darle, Margot Jarrige, Hélène Polveche, Lina El Kassar, Séverine Julié, Sandrine Bessou-Touya, Nathalie Holic, Gilles Lemaitre, Cécile Martinat, Christine Baldeschi, Jennifer Allouche

**Affiliations:** 1 Centre d’Etude des Cellules Souches, Institute for Stem Cell Therapy and Exploration of Monogenic Diseases, French Muscular Disease Association, 91100 Corbeil-Essonnes, France; 2 Centre d’Etude des Cellules Souches, Institute for Stem Cell Therapy and Exploration of Monogenic Diseases, Cell Biology Platform, Research and Technological Innovation Team, 91100 Corbeil-Essonnes, France; 3 Pharmacology Unit, Pierre Fabre Dermo-Cosmétique, 31100 Toulouse, France; 4 INSERM U861, Institute for Stem Cell Therapy and Exploration of Monogenic Diseases, 91100 Corbeil-Essonnes, France; 5 U861, Evry Val d’Essonne/Paris-Saclay University, 91100 Corbeil-Essonnes, France

**Keywords:** skin regeneration, pigmentation-related skin disorders, human pluripotent stem cell

## Abstract

Melanocytes are essential for skin homeostasis and protection, and their loss or misfunction leads to a wide spectrum of diseases. Cell therapy utilizing autologous melanocytes has been used for years as an adjunct treatment for hypopigmentary disorders such as vitiligo. However, these approaches are hindered by the poor proliferative capacity of melanocytes obtained from skin biopsies. Recent advances in the field of human pluripotent stem cells have fueled the prospect of generating melanocytes. Here, we have developed a well-characterized method to produce a pure and homogenous population of functional and proliferative melanocytes. The genetic stability and potential transformation of melanocytes from pluripotent stem cells have been evaluated over time during the in vitro culture process. Thanks to transcriptomic analysis, the molecular signatures all along the differentiation protocol have been characterized, providing a solid basis for standardizing the protocol. Altogether, our results promise meaningful, broadly applicable, and longer-lasting advances for pigmentation disorders and open perspectives for innovative biotherapies for pigment disorders.

## 1. Introduction

Melanocytes are pigment-producing cells derived from the neural crest, a transient embryonic multipotent stem cell population that emerges from the neural plate in vertebrates [1]. Defects in the development or the homeostasis of melanocytes are present at the onset of many pigmentary disorders such as vitiligo, for which therapeutic solutions remain a challenge [2]. Surgical therapy consists of the re-introduction of melanocytes in depigmented lesions. Primary melanocytes can be isolated from pigmented skin of the same person by different procedures (e.g., tissue grafts or cellular grafts) [3]. However, these techniques require surgical biopsy and can induce the depletion of the site donor. In addition, cells derived from primary culture have a limited life span [4]. To overcome these limitations, human pluripotent stem cells (hPSCs) have become a promising alternative as an unlimited source of melanocytes. In recent years, various protocols have been reported to successfully convert hPSCs into melanocytes [5,6,7,8,9,10]. Notwithstanding this progress, the generation of hPSC-derived melanocytes still relies on the enrichment of the desired cell population by cell sorting or mechanical selection. In addition, the proliferative status of hPSC-derived melanocytes and their maintenance during long periods of culture has not been extensively investigated. Here, we develop a multi-step protocol for specifying hPSCs into a homogeneous population of melanocytes without any mechanical or cell-sorting steps. These hPSC-derived melanocytes, generated from the two origins of hPSCs, human embryonic stem cells and human-induced pluripotent stem cells, are suitable for long-term expansion without losing their phenotypes and their functional capacity to integrate 3D in vitro and in vivo skin equivalents when cocultured with primary fibroblasts and primary keratinocytes.

## 2. Results

### 2.1. Characterization of a Pure and Homogenous Population of Melanocytes Derived from hPSC

**Figure 1:**
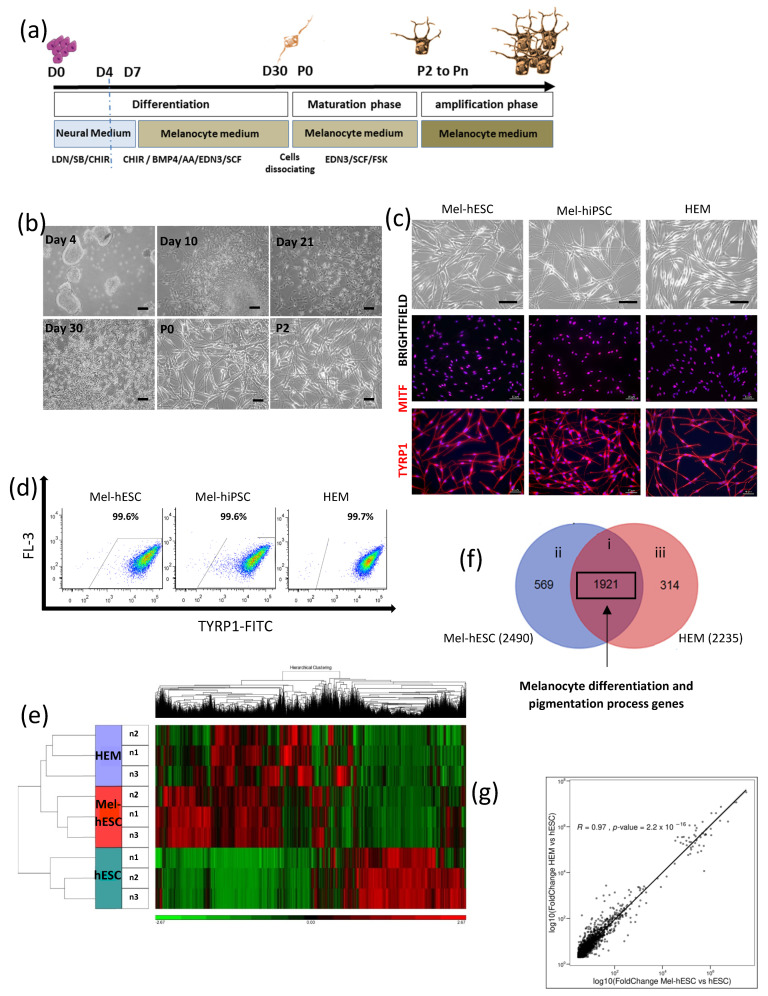
Characterization of hPSC-derived melanocytes. (**a**) Diagram of the melanocytic differentiation from hPSCs. Illustrated using Servier Medical Arts-SMART image bank. (**b**) Microscopy images at different stages of melanocytic differentiation from hPSCs (scale bar: 50 µm). (**c**) Microscopy images of Mel-hESC and Mel-hiPSC at passage 6 (P6) compared to HEMs (P6). Immunofluorescence staining of MITF and TYRP1 in Mel-hPSC (scale bar: 50 µm). (**d**) Flow cytometry analysis of TYRP1 in Mel-hESC (P7), Mel-hiPSC (P6), and HEMs (P6). The Mel-hESC value represents the mean of six independent experiments with an SD ± 0.4. The HEM value represents the mean of three independent experiments with an SD ± 0.1. (**e**) Hierarchical clustering of gene expression. Comparison between HEMs (P6), Mel-hESC (P4), and hESC (*n* = 3). (**f**) Venn diagram showing comparative gene expression profiles of Mel-hESC (P4) and HEMs (P6). Gene lists are defined as genes upregulated in the two cell lines greater than 2-fold and with an FDR < 0.001 compared to hESCs. i represents common list between Mel-hESC and HEMs, ii as the 569 uregulated genes specific to Mel-hESC and iii as the 314 genes specific to HEMs. (**g**) Scatterplot showing a high correlation of 1921 gene expression levels in HEMs and Mel-hESC (R = 0.967) from indicated regions (i) of Venn diagram defined in (**f**).

To reproduce the development of the melanocyte chronobiology, we have established a multistep protocol in which human-induced pluripotent stem cells (hiPSCs) and human embryonic stem cells (hESCs) are differentiated into melanocyte precursors by the induction of the neural crest lineage followed by a maturation step (Figure 1a). First, hPSCs were aggregated in embryoid bodies (EBs). The induction of hPSCs within the neural crest lineage was initiated by the use of dual-SMAD inhibition in combination with Wnt signaling [11,12]. At day 4, and to initiate the differentiation into melanocytes, small molecules including endothelin-3 (EDN3), stem cell factor (SCF), bone morphogenetic 4 (BMP4), and ascorbic acid (AA) were added at specific concentrations [7]. At day 7, EBs were placed on gelatin and grown in the melanocyte medium (MGM-4) supplemented by the same molecules. In these conditions, cells migrated out of the EBs. From day 21 to day 30, cells acquired a melanocyte-like dendritic cell morphology. The induction of melanocyte maturation was performed at day 30. For this purpose, cells were first dissociated with trypsin and plated in MGM-4 supplemented with EDN3, SCF, and forskolin (FSK), essential for the survival and maturation of melanocytes. After two passages, melanocytes with a typical mature morphology were observed (Figure 1a,b). FSK was added to the medium in addition to the EDN3 and SCF to induce the maturation from the cell dissociation at day 30 corresponding to passage 0 (P0) up to passage 2 (P2). Then, the melanocyte population (Mel-hPSC) was maintained in MGM-4 medium along the amplification phase and exhibited a typical morphology of human melanocytes (Figure 1c). As a positive control, human epithelial melanocytes (HEMs) were used for further characterization. Analysis by immunofluorescence staining for melanocytic markers indicated that most of the Mel-hiPSC and Mel-hESC expressed MITF (microphthalmia-associated transcription factor), which is considered the main transcription factor of melanogenesis as well as its targets, such as TYRP1 (tyrosinase-related protein 1) (Figure 1c). This result was confirmed by flow cytometry analysis revealing that more than 99% of cells were positive for TYRP1 expression (Figure 1d). A transcriptomic analysis was performed to analyze the gene expression profile of Mel-hESC in comparison with HEMs. Hierarchical clustering showed similar gene expression profiling between Mel-hESC and HEMs compared to hESCs (Figure 1e). Almost 70% of genes upregulated in comparison to hESCs were found to be common between Mel-hESC and HEMs (Figure 1f). Gene ontology analysis of these common genes revealed a close genetic signature with biological processes related to “melanocyte differentiation” and the “pigmentation process” (Figure 1f and Figure S1(i)). Moreover, the gene expression levels were highly correlated between patient iMels and HEMs (R = 0.97) (Figure 1g). These results demonstrated the generation of a pure population of melanocytes.

### 2.2. Functional Characterization of hPSC-Derived Melanocytes

**Figure 2:**
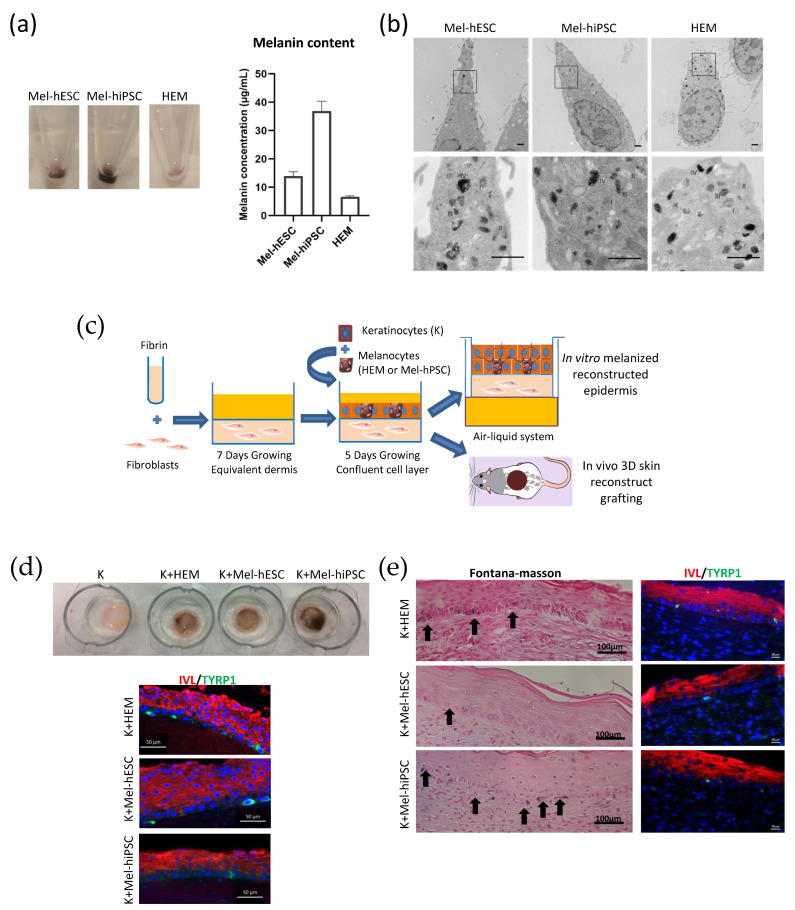
Functionality of hPSC-derived melanocytes. (**a**) (left) Cell pellet pictures of Mel-hESC, Mel-hiPSC at passage 7, and HEMs. (right) Melanin content measurement in Mel-hESC, Mel-hiPSC, and HEMs. Each value represents the mean of three independent experiments. (**b**) Representative electronic microscopy images of melanosome stages in Mel-hESC, Mel-hiPSC, and HEMs. Characteristic immature unpigmented (stages I/II) and mature pigmented (stages III/IV) melanosomes are observed in the soma of melanocytes (scale bar: 10 μm). (**c**) Experimental diagram of in vitro pigmented epidermis reconstruction and in vivo grafting. Illustrated using Servier Medical Arts-SMART image bank. (**d**) Analysis of the in vitro pigmented reconstructed epidermis. (Upper) Inset pictures containing pigmented reconstructed epidermis without melanocytes (K), containing HEMs (K + HEMs), Mel-hESC (K + Mel-hESC), or Mel-hiPSC (K + Mel-hiPSC). (Lower) Immunofluorescence staining of Involucrin (IVL) and TYRP1 on sections of the reconstructed epidermis containing HEMs, Mel-hESC, or Mel-hiPSC (scale bar: 50 μm). (**e**) Fontana–Masson (left) stain and Involucrin (IVL) and TYRP1 immunofluorescence staining (right) on regenerated skin sections containing HEMs, Mel-hESC, or Mel-hiPSC. Black arrows show melanin staining in HEMs, Mel-hESC, or Mel-hiPSC integrated into the basal layer of regenerated skin (scale bar: for Fontana Masson (FM), 100 μm; for Immunofluorescence (IF), 50 μm). The Mel-hPSC used for the evaluation of functionality is at passage 6. Red color is Involucrin stain and green color represent TYRP1 stain.

The functionality of melanocytes derived from hPSCs was first assessed by the quantification of the pigmentation in cell pellets by using spectrophotometry analysis. The quantification of the melanin content demonstrated the capacity of mel-hiPSC and mel-hESC cells to synthesize melanin (Figure 2a). Electron microscopy analysis also showed the presence of the four maturation stages of melanosomes localized in the cytoplasm of Mel-hPSC as observed in HEMs (Figure 2b). Finally, we evaluated the capacity of Mel-hPSC to integrate into an in vitro and in vivo 3D reconstructed skin. For this purpose, primary human keratinocytes (K) were added to the equivalent dermis with HEMs or Mel-hPSC. They were expanded until confluency and were then maintained at the air–liquid interface in vitro for 14 days or grafted in vivo onto nude mice for two weeks (Figure 2c). Macroscopic analysis revealed a well-pigmented epidermis under in vitro coculture conditions using Mel-hPSC and K comparable to those observed in the HEM and K conditions (Figure 2d). The presence and distribution of melanocytes and keratinocytes were confirmed by immunostaining for involucrin (IVL) in keratinocytes and TYRP1 in melanocytes (Figure 2d). Their localization was also confirmed by additional immunostaining for Keratin K14 (KRT14) and TYROSINASE (TYR) (Figure S2). In parallel, the 3D reconstructed skin in vivo showed a normal tissue architecture two weeks after grafting. As shown by the Fontana-Masson coloration, Mel-hPSC produced melanin. Immunostaining analysis also revealed the expression of TYRP1 as well as the localization of the mel-hiPSC and mel-hESC in the basal layer of the epidermis (Figure 2e). Altogether, these data demonstrated the high functionality of Mel-hPSC.

### 2.3. Long-Term Expansion of hPSC-Derived Melanocytes and Genomic Integrity

**Figure 3:**
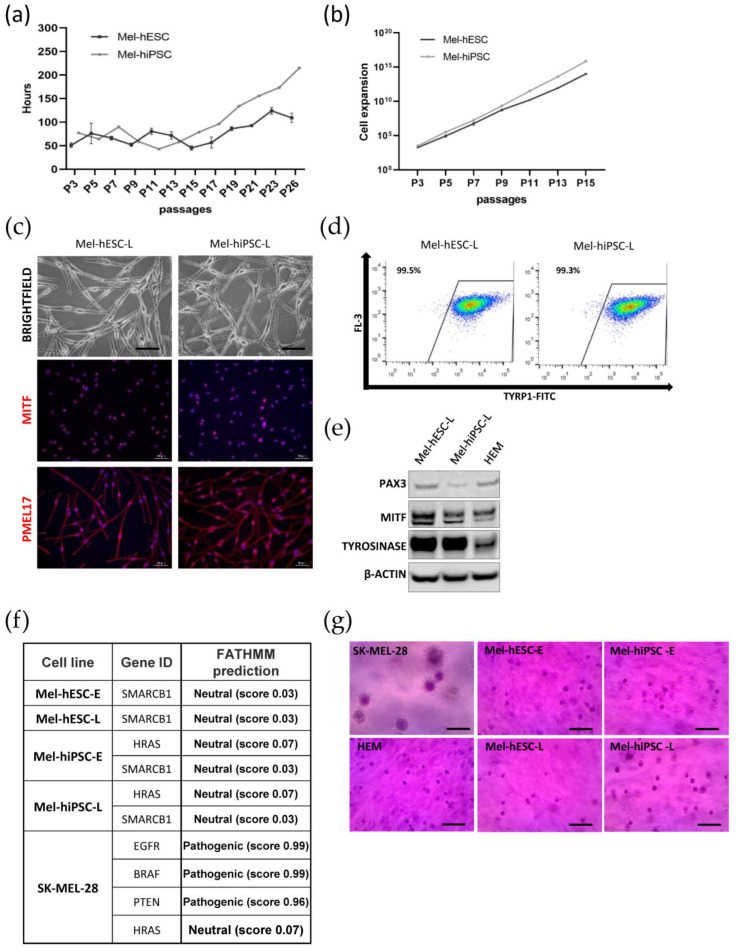
Long-term proliferation of hPSC-derived melanocytes. (**a**) Doubling time measurements during the growing phase of the Mel-hESC and Mel-hiPSC, from passage 3 (P3) to passage 26 (P26). (**b**) Cumulated cell expansion calculated from passage 3 (P3) to passage 15 (P15). For the next parts of the figure, Mel-hPSC early passages (Mel-hPSC-E) are defined from P3 to P15 and Mel-hPSC late passages (Mel-hPSC-L) are defined from P15 to P26. (**c**) Microscopy analysis and immunofluorescence analysis of MITF, TYRP1, and PMEL17 in Mel-hESC-L and Mel-hiPSC-L. Scale bar is 50 µm. (**d**) Flow cytometry analysis of TYRP1 in Mel-hESC-L and Mel-hiPSC-L. Mel-hESC-L value represents the mean ± SD 0.05 of three independent experiments. (**e**) Expression of PAX3, MITF, and TYROSINASE proteins by Western blot in Mel-hESC-L and Mel-hiPSC-L compared to HEMs. Band intensities are normalized to β-actin. (**f**) AmpliSeq Cancer Hotspot Panel Library v2 in Mel-hESC and Mel-hiPSC at early and late passages. Samples tested included melanoma cell line (SK-MEL-28) as a positive control. The SNPs detected are compared to the database FATHMM (http://fathmm.biocompute.org.uk, accessed on 6 March 2017). (**g**) Soft agar assay for colony formation on early and late passages of Mel-hESC and Mel-hiPSC. SK-MEL-28 is used as a control for the melanoma cell line. HEMs are used as a control for adult melanocytes (scale bar is 50 µm).

Next, we investigated the proliferative potential of Mel-hPSC by measuring the doubling time at early (from passage 3 to passage 15) and late passages (>passage 15) (Figure 3a). At early passages, both Mel-hESC and Mel-hiPSC maintained a stable doubling time between 50 and 80 h. At later passages, an increased doubling time over 100 h was observed. At passage 26, the doubling time reached 200 h and 120 h for Mel-hiPSC and Mel-hESC, respectively (Figure 3a). By determining the cumulative cell expansion for Mel-hPSC until P15, our results demonstrated the possibility of generating a large-scale bank of approximately 10^14^ cells (Figure 3b). Furthermore, we showed that over time in culture, Mel-hPSC maintained their dendritic morphology (Figure 3c) and expressed all the melanocytic markers, as shown by immunostaining (Figure 3c), flow cytometry (Figure 3d) and immunoblot analysis (Figure 3e). We next evaluated the genomic integrity of Mel-hPSC maintained for a long period in culture. The karyotyping of Mel-hPSC at late passages showed no chromosomal abnormalities (Figure S3). We also sought to analyze the appearance of mutations of 50 oncogenic and tumor suppressor genes using the AmpliSeq Cancer Hotspot Library v2 (Thermo Fisher Scientific: Waltham, MA, USA) in Mel-hPSC at early and late passages. We used one melanoma cell line (SK-MEL-28 from ATCC) carrying the pathogenic mutations in EGFR, BRAF, and PTEN *(*https://maayanlab.cloud/SK-MEL-28, accessed on 10 January 2023) as a positive control. The presence of single nucleotide polymorphisms (SNP) was also analyzed in Mel-hPSC, revealing the appearance of neutral mutations as referred to by the database FATHMM (http://fathmm.biocompute.org.uk, accessed on accessed on 6 March 2017). Altogether, these results indicated the absence of a genomic tumor in the Mel-hPSC at late passages. To complete this genomic analysis, a soft agar colony formation was assessed on Mel-hPSC at early and late passages to evaluate their potential malignant transformation. While large aggregates were observed with the SKMEL28 melanoma cell line, no aggregate was detected with Mel-hPSC and HEMs. These results showed that over time in culture, the Mel-hPSC is not transformed (Figure 3g).

### 2.4. Functional Genomic Analysis of Melanocyte Differentiation and Maturation Processes

**Figure 4:**
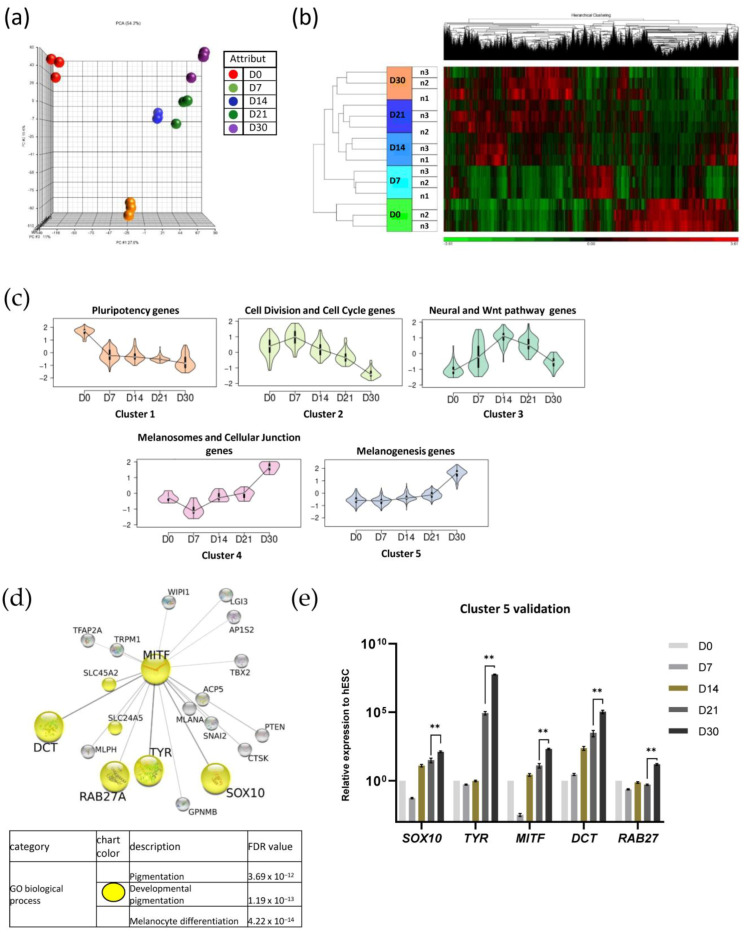
Transcriptional analysis of Mel-hESC during the differentiation process in a time-dependent manner. (**a**) Principal components analysis (PCA) of each differentiation time point (day 0: D0, day 7: D7, day 14: D14, day 21: D21, and day 30: D30). (**b**) Hierarchical clustering of gene expression at D0, D7, D14, D21, and D30 (*n* = 3). (**c**) Graphics of 5 clusters. Clustering of genes statistically differentially expressed during differentiation and regrouped with the same expression profile. (**d**) (Upper) Gene network linked to MITF. The 328 genes contained in cluster 5 are analyzed in STRINGdb (with medium confidence 0.4). The global network obtained is sent to Cytoscape to identify a gene sub-network directly linked to MITF. A score is attributed to edges in terms of connectivity with MITF [13]. The size of gene circles is correlated with these scores. Genes circles colored in yellow are members of the “developmental pigmentation” gene ontology biological process term. (Lower) Top gene ontology biological process terms from enrichment analysis of the global STRINGdb network. Quantitative PCR of *SOX10*, *MITF*, *DCT*, *TYR*, and *RAB27A* during hESC differentiation from D0 to D30. The data are normalized to 18S and expressed as relative expressions of undifferentiated hESCs at D0. (**e**) Cluster 5 validation. Quantitative PCR of *SOX10*, *TYR*, *MITF*, *DCT* and *RAB27* for Mel-hESC from D0 to D30. The data are normalized to 18S and expressed as relative expressions of D0. ** *p* < 0.01.

**Figure 5:**
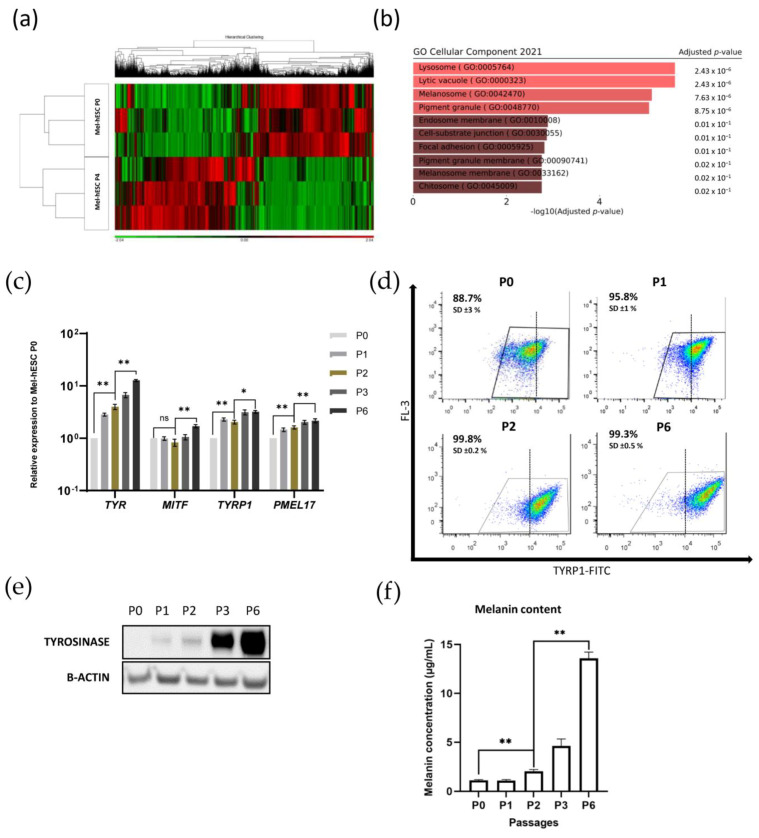
Maturation phase of hPSC-derived melanocytes. (**a**) Hierarchical clustering of gene expression. Comparison of Mel-hESC at passage 0 (P0) and Mel-hESC at passage 4 (P4) (*n* = 3). P0 is the first passage after the dissociating stage after 30 days of differentiation. (**b**) Gene ontology enrichment analysis of 1309 upregulated DEGs. Comparison of gene expression of Mel-hESC at P4 to P0 via ENRICH’R tool. (**c**) Quantitative PCR of *MITF*, *TYR*, *TRP1*, and *PMEL17* for Mel-hESC from P0 to P6. The data are normalized to 18S and expressed as relative expressions of Mel-hESC at P0. (**d**) TYRP1 expression by flow cytometry analysis of Mel-hESC at P0, P1, P2, and P6. Each value represents the mean of three independent experiments with SD. The dotted line represents a TYRP1 intensity threshold established on cells at P6. (**e**) Expression analysis of tyrosinase (TYR) by Western blot in Mel-hESC P0, P1, P2, P3, and P6. Band intensities were normalized by β-actin. (**f**) Melanin content measurement in Mel-hESC P0, P1, P2, P3, and P6. Each value represents the mean ± SD from three independent experiments. For statistical significance, n.s. denotes nonsignificant, * *p* < 0.05 and ** *p* < 0.01.

We next sought to determine the molecular pathways that drive melanocyte differentiation by using next-genome sequencing (NGS) technology and differential expression of genes (DEG) analysis. The expression pattern of the whole genome was analyzed in a time-dependent manner at day 0, day 7, day 14, day 21, and day 30 of the differentiation process. Principal component analysis (PCA) showed a similar transcriptional profile between replicates of each time point, demonstrating the robustness of the protocol (Figure 4a). This observation was supported by the hierarchical clustering overview of the transcriptomic analysis (Figure 4b). To decipher the molecular and cellular pathways involved in each stage of the developmental process, we performed a kinetic analysis on differentially expressed genes for every comparison (respectively, day 0 versus day 32, day 7 versus day 32, day 14 versus day 32, and day 21 versus day 32). Genes were regrouped in eight clusters according to the kinetics of their differential expression (Table 1). Five clusters have been analyzed by gene ontology using the ENRICH’R interface (Figure S4). These five clusters are shown in Figure 4c. Cluster 1 included genes primarily related to the pluripotency, morphogenesis, and development of epithelial tissues. Cluster 1 was gradually repressed from day 0 to day 30. In the second cluster, the expression of genes associated with cell division and the regulation of the cell cycle increased from day 0 to day 7 and then decreased as the cells migrated out of the EBs. Cluster 3 is composed of several genes involved in the Wnt signaling pathway and in the negative regulation of embryonic development and neurogenesis. Their expression gradually increased from day 0 to a peak on day 14. The molecular signaling pathway involved in melanogenesis is grouped into two clusters (4 and 5) and included 338 genes. These genes showed a differential expression between day 21 and day 30. Functional enrichment analysis on cluster 5 revealed 19 genes directly connected to *MITF*, the master regulator of melanogenesis, among which 6 genes including *SOX10*, *DCT*, *TYR*, and *RAB27* (yellow circles in Figure 4d) are defined as being involved in “developmental pigmentation” (Figure 4d). To confirm our data, the expression profiles of these markers were validated by quantitative real-time PCR (Figure 4e). While all of this molecular characterization was performed during the differentiation processes, we next sought to molecularly define the maturation step. We thus compared gene expression profiles between P0 and P4, which are immature and mature Mel-hESC, respectively. These transcriptomic analyses showed a differential gene expression pattern at P4 when compared to P0 (Figure 5a). Many differentially expressed genes (DEGs) were identified (*p*-value adjusted ≤ 0.5; fold change ≥ 1.5; minReads100) with 1309 upregulated and 1719 downregulated DEGs. Gene ontology analysis of the upregulated DEGs revealed biological processes associated with “lytic vacuole”, “lysosome”, and “melanosome” (Figure 5b). Gene expression analysis for *MITF* and its downstream target genes (*TYPR1*, *TYR*, and *PMEL17*) in immature Mel-hESC at P0 to the mature P6 stage revealed an increase in melanogenesis genes (Figure 5c). Flow cytometry analysis also showed an increase in the level of TYRP1 expression with the passages of Mel-hESC (Figure 5d). Similarly, Western blot analysis indicated that the major tyrosinase enzyme involved in melanin synthesis was not expressed at passage 0 and became progressively expressed after several passages (Figure 5e). Accordingly, a progressive increase in melanin synthesis was observed from passage 2 to 6 (Figure 5f). Thus, our data indicated the acquisition of a mature phenotype of Mel-hESC with the passages.

## 3. Discussion

In this study, we provide a multi-stage protocol for generating a pure and homogenous population of functional melanocytes derived from human pluripotent stem cells associated with a description of the molecular signatures during the differentiation and maturation processes.

Our method is based on a multi-step protocol which includes a neural crest induction phase followed by sequential phases of differentiation, maturation, and amplification. This temporality led to the generation of mature and functional melanocytes from both the human embryonic stem cell and human-induced pluripotent stem cell lines, with equal efficacy. We based our approach on the temporal regulation of a combination of cytokines and growth factors at specific concentrations. To obtain an efficient population of neural crest cells in 4 days, we used dual-SMAD inhibition in combination with a Wnt signaling activator in a neural medium. To direct the neural crest cells into melanocytes, we used SCF, EDN-3, and Chir99021, a Wnt activator. This combination of molecules was described as necessary for melanocyte development and survival [14,15]. In addition to these molecules, and based on our previous works [7,16], BMP4 and AA at specific concentrations were added to generate melanocytes after 30 days of differentiation. A major difference in our approach compared to previously published protocols lies in the sequential use of one of the most potent cAMP inducers, the forskolin. In our method, cells were treated with forskolin only during the maturation phase. This activation leads to the transcription of MITF, which in turn regulates the principal enzyme of melanin production [17,18]. In 7 to 10 days, maturation of the unpigmented melanocytes was observed by the acquisition of a dendritic morphology, which is characteristic of the primary human melanocytes as well as by their ability to synthesize melanin. These results suggest that forskolin may act as a maturation agent. Another important difference resides in the fact that our approach is not based on manual selection or FACS, as is the case in a number of previously published studies [7,10,19]. These techniques are time consuming, experimenter dependent, and can lead to high variability. With the sequential addition of specific cytokines mimicking the chronobiology of melanocyte development, we obtained, from day 30, a pure population of unpigmented cells stained positive for the melanocytic enzyme TYRP1 at more than 90% but negative for Tyrosinase, the main enzyme of the melanin synthesis. Tyrosinase expression is observed after 7–10 days in the presence of forskolin, correlating with melanin production. Altogether, our results demonstrated the possibility to generate, without any selection, a pure population of a precursor of melanocytes capable of maturing in the presence of cAMP inducers.

Pigmented melanocytes can then be easily amplifiable in a medium suitable for the long-term culture of melanocytes. At the functional level, our results demonstrated that mel-hESC and mel-hiPSC are capable of producing melanin, synthesizing melanosomes, and integrating into an in vitro reconstituted pluristratified epidermis. In addition, in vivo experiments have demonstrated their ability to move to the basal layer of the epidermis when incorporated into immunodeficient mice. Overall, these experiments demonstrate the production of highly functional mature melanocytes.

To date, most of our knowledge about the molecular mechanisms involved in melanogenesis has been derived from experiments in mice [20]. Due to architectural and functional differences between human and mouse skin, extrapolation of results from mouse to human remains limited. Thanks to a transcriptomic approach, five different clusters of genes have been highlighted as representative of the developmental stages of human melanocytes. Several clusters of genes have been identified, including the important canonical Wnt signaling pathway, that play a role in neural crest induction [21], and then in melanocytic lineage induction [1]. Furthermore, the differentiation process in melanocytes was associated with a signature of genes related to the melanogenesis process, which demonstrated the efficiency of the protocol [22]. In addition, to characterize the maturation process, the gene expression profile of mature (pigmented) melanocytes was compared with immature (non-pigmented) melanocytes. We identified well-known genes upregulated in mature melanocytes such as Tyrosinase and TYRP1, the main melanogenesis enzymes [23]. Our analysis revealed additional genes involved in the biological processes of melanosomes, lysosomes, and pigment granules. Interestingly, several of these genes encode for different isoforms of the RAB family members. Functional impairment of RAB proteins such as Rab27A has been reported to cause pigmentation defects [24]. Our analysis revealed the differential expression of RAB isoforms in mature melanocytes compared to the immature population. Although speculative at this stage, further experiments could be envisaged to evaluate their defects in melanosome biogenesis and transport. The molecular characterization of the maturation step provides new tools to study different stages of human melanocytes. Altogether, our results showed a well-characterized sequential protocol that allowed the generation of melanocytes from hPSCs through the neural crest induction followed by the generation of melanocyte precursors and a maturation step into functional melanocytes. Apparition of specific markers at a specific time point led to a molecular signature, allowing the molecular qualification of the defined steps from the neural crest induction to the melanocyte speciation. These findings will help to standardize the protocol.

Another advantage of the method described in this study resides in the long-term stability of produced melanocytes without the loss of their phenotype. This work opens new perspectives in terms of cell therapy for patients with pigmentation defects. The most widespread acquired depigmentation disorder is vitiligo. No known treatment can consistently induce repigmentation in all patients [25], and for severe vitiligo, the only option is surgery [26]. The efficacy of surgical methods has always been variable, and this technique can cause the depletion of donor sites. The life span of adult melanocytes after transplantation also seems to be limited to less than one year, which limits the treatment possibilities both at the surface and in the number of re-applications. Thus, the therapeutic potential of hiPSCs in autologous transplantation appears to be a promising option. To date, the capacity of melanocytes derived from human pluripotent stem cells to be maintained long term in culture has not been fully investigated. It has been previously reported that the cell proliferation capacity of Mel-hPSC decreases with passages, and after several generations in culture, cells begin to slowly proliferate and differentiate [4]. In our previous published protocol, we isolated melanocytes and amplified them for up to 12 passages [7]. More recently, Cohen et al. demonstrated the proliferative activity of melanocytes derived from different donors during sub-culture from passage 6 to passage 12. In this study, we increased the purity of differentiated populations, which is important to warrant safety prior to clinical cell therapies and show the capacity to generate a highly effective functional melanocyte. We characterized the genomic stability, and potential transformation during the in vitro process of melanocytes at low and late passages was evaluated. The newly melanocytes were able to be amplify for up to 26 passages (more than 250 days).

For the purpose of cell therapy, a number of tests verifying the level of tumorigenicity, distribution, and immunogenicity will be necessary [27]. Recent studies showed that autologous transplantations of iPSC-derived cells were performed with no serious adverse events noted [27,28]. In this study, we evaluated whether over time in culture, mel-CSP may lead to genomic mutation and tumor formation. To accomplish this, we tested, at early and late passages, the most common genes, including HRAS, NRAS, and BRA, known to induce early-stage melanoma transformation and which are not mutated in mel-CSP. There was also no transformation observed over time in culture. For the purpose of cell therapy, the risk of melanomagenesis after transplantation must be further evaluated by reglementary toxicology studies in animal models. Liu et al. showed the absence of tumorigenicity 7 weeks post transplantation of mel-iPSC. In their model, the mel-iPSC from vitiligo patients was injected into the back skin of nude mice and mixed with dermal fibroblasts and epidermal cells isolated from the skin of neonatal mice [29]. However, one of the most important factors known to activate melanomagenesis is ultraviolet light [30,31]. Although narrow-band UV-B phototherapy is used in the context of autologous melanocyte cell transplantation to enhance pigmentation with no side effects reported [3,32,33], additional UV exposure experiments of mel-CSP must be assessed before grafting.

In addition, an important mechanism of vitiligo pathogenesis is a CD8 T cell-mediated autoimmune disease hypothesis. Antigen-specific T-cell reactivity to HLA-A2-restricted melanocyte epitopes, which includes gp100, tyrosinase, and melanA/MART, was detected [30,31,34,35]. While the HLA-A subtype is expressed in 35 to 45% of the population [31], an increase in HLA-A2-positive results has been observed in most vitiligo patients [36,37]. Furthermore, a positive correlation between vitiligo disease activity and reactivity to the melanocyte antigen gp100 has been described [38]. One possible way to avoid the destruction of melanocytes derived from iPSC transplantation could be to combine cell-based therapy with antagonist peptide ligands that might block specific T-cell responses.

Overall, the development of a well-characterized system to generate melanocytes from hPSC will serve to improve the knowledge of the mechanisms involved in the onset of pigmentation disorders and may open up new possibilities for cell-based therapeutic applications.

## 4. Materials and Methods

Cell culture. hESCs from one cell line, SA-01 (Cellartis, Götenborg, Sweden), and hiPSCs from one cell line 207c02 were reprogrammed from dermic fibroblasts and grown as previously described [16]. For the differentiation, embryonic bodies (EBs) were formed from hESCs and hiPSCs. Briefly, after a first trypsinization with trypsine/EDTA 0.05% (ThermoFisher, Waltham, MA, USA) for 2–3 min to eliminate the feeder cells, Stempro Accutase cell dissociation reagent (ThermoFisher) was applied for 1–2 min to detach hPSCs in small clumps. Cells were subjected to spinning for 3 min at 110× *g* and delicately resuspended to avoid the formation of single cells. Cells were grown on low-attachment dishes in a neural medium composed of neurobasal and Ham’s F12 (ratio 1:1) complemented with 2% of B-27 without vitamin A and with 1% of N-2 (all from ThermoFisher). Neural crest induction was performed by using CHIR-99021(3 µM) (TOCRIS), LDN-193189 (0.2 µM) (Miltenyi Biotec, Bergisch Gladbach, Germany), and SB 431542 (40 µM) (TOCRIS). EBs were grown in the same medium for 4 days. At day 4, the neural medium was supplemented with 3 µM CHIR99021, 100 nM EDN3 (American peptide, Albuquerque, NM, USA), 50 ng/mL SCF (Peprotech, NJ, USA), 0.02 nM of human recombinant BMP4 (Peprotech, Cranbury, NJ, USA), and 0.3 mM ascorbic acid (Sigma-Aldrich, St. Louis, MO, USA). From day 7 to day 30, EBs were plated on gelatine 0.1% (Sigma-Aldrich) and grown in MGM-4 medium (MGM-4 BulletKit Lonza, Allendale, NJ, USA) supplemented with 0.5 µM CHIR99021, 100 nM EDN3, 50 ng/mL SCF, 0.02 nM of human recombinant BMP4, and 0.3 mM ascorbic acid. The medium was changed every 2–3 days. Cells were dissociated using trypsin 0.05% and grown in the same medium supplemented with 20 µM forskolin (Sigma-Aldrich, St. Louis, MO, USA) during 2 passages. For passage 2, MGM-4 medium was used without any supplements to culture the pigmented melanocytes. Cells were passaged at least 20 times. The melanoma cell line SK-Mel-28 (HTB-72™, ATCC, St, Washington, DC, USA) was grown in DMEM F12 medium supplemented with 10% FBS (all from ThermoFisher).

Quantitative RT-PCR (QRT-PCR). Total RNA was isolated from hESCs, hiPSCs, and HEMs using RNeasy plus Mini extraction kit on Qiacube (Qiagen, Hilden, Germany) including an on-column DNase digestion step according to the manufacturer’s protocol. The total RNA was isolated and quantified using a Nanodrop 2000 spectrophotometer (ThermoFisher, Waltham, MA, USA). A measure of 500 ng of total RNA was used for reverse transcription using the Superscript III reverse transcription kit (ThermoFisher). QRT-PCR analysis was performed using a QuantStudio™ 12 Flex instrument device and Luminaris Color HiGreen QRT-PCR Master Mixes Low Rox (ThermoFisher) following the manufacturer’s instructions. The PCR amplification process comprised 40 cycles of denaturation at 95 °C for 10 s, annealing at 55 °C for 30 s, and extension at 95 °C for 5 s. The quantification of gene expression was based on the ΔCt Method and normalized on 18S expression. QRT-PCR was performed using the primers described in Table 2.

Immunocytochemistry. Cells were seeded on glass slides and fixed in 4% paraformaldehyde (Sigma-Aldrich) at RT for 10 min. After three washes, cells were permeabilized with 0.1% Triton X-100 (Sigma-Aldrich) in PBS for 5 min and blocked with 1% BSA (Sigma-Aldrich) in PBS for 1 h at RT. Primary antibody incubation was carried out overnight in blocking solution at 4 °C, 1:200 mouse anti-MITF (DAKO, Santa Clara, CA, USA; M3621), 1:100 mouse anti-TYRP1 (Lsbio, Seattle, WA, USA; LSC39939), and 1:100 mouse anti-PMEL17 (Abcam, Cambridge, UK; ab15228). Secondary antibody 1:1000 goat anti-mouse 555 (ThermoFisher, A-21422) was applied together with DAPI 1:10,000 (ThermoFisher, D1306) in PBS for 1 h at RT. After triple washing, slides were mounted in Fluoromount-G™ Mounting Medium (ThermoFisher, 00-4958-02). Images were captured using a epifluorescence illumination microscope (Axio Imager, Zeiss) and Zen 2 3.2 (blue edition) software.

Flow cytometry. Cells were fixed and permeabilized in FCM permeabilization buffer (Santa-Cruz biotechnology, Dallas, TX, USA) at RT for 4 min. Cells were then washed with PBS and incubated at RT for 1 h in 0.1% FBS with 1/100 dilution of mouse anti-TYRP1 antibody conjugated AF488 (Novusbio, Littleton, CO, USA; NBP2-34720). Isotype-specific IGg2a-AF488 was used as a control (Novusbio; ICC003G). Data were acquired on the MACSQuant Analyzer 10 (Miltenyi Biotech, Bergisch Gladbach, Germany) and analyzed using FlowJo v10 software (FlowJo LLC, Ashland, OR, USA).

Western blotting. Cell pellets were lysed in RIPA lysis buffer (Sigma-Aldrich) supplemented with anti-proteases (Sigma-Aldrich) and anti-phosphatase (Roche, Indianapolis, IN, USA). Protein concentrations were quantified by pierce BCA protein assay (ThermoFisher). Western blotting was performed by standard techniques using 4–12% NuPAGE Novex or 3–8% Bis-Tris gels (ThermoFisher, Waltham, MA, USA) and nitrocellulose membranes (Whatman, GE Healthcare, Chicago, IL, USA). Blots were blocked in 5% milk in PBS 0.1% Tween 100 (Sigma-Aldrich), incubated with 1/500 of anti-Pax3 (DSHB, Iowa City, IA, USA), 1/500 of rabbit anti-MITF (ab20663, Abcam, Cambridge, UK), and 1/1 000 of mouse anti-Tyrosinase (ab738, Abcam), followed by 1:10,000 of secondary antibody anti-HRP. Quantitative normalization was achieved with incubation with 1/50,000 β-actin-HRP (A-3854, Sigma-Aldrich). Immunoreactive bands were revealed using Amersham ECL Plus Western Blotting Detection Reagents (GE Healthcare, Chicago, IL, USA). The ImageQuant LAS 4000 mini (GE Healthcare, Chicago, IL, USA) s used for image capturing and analysis with the software ImageQuant LAS 4000 v1.1.

Organotypic cultures and grafting onto mice. The organotypic epidermis was generated as previously described [16]. Briefly, bioengineered skin equivalents were generated with a fibrin matrix [mixture of human plasma, given by J.-J. Lataillade at the Biomedical Research Institute of French Armies (INSERM U1197, Clamart, France) and saline solution composed of sodium chloride, calcium chloride, and Exacyl (Sanofi, Paris, France)] populated with human neonatal fibroblasts (Promocell). Primary keratinocytes (CELLnTEC, Bern3/23/23 3:43:00 PM, Switzerland) and HEM/Mel-hESC/Mel-hiPSC with a ratio of 1:1 were seeded on the fibrin matrix, grown immersed to confluence for 10 days in FAD medium (3:1 mixture of Dulbecco’s modified Eagle’s medium [DMEM] and Ham’s F12 media, ThermoFisher) supplemented with SCF (10 ng/mL), EDN3 (100 nM), FSK (20 µM), and αMSH trifluoroacetate 0.1 µM (Sigma-Aldrich). One part of the bioengineered skin equivalents was transferred into PET inserts (BD Falcon, Franklin Lakes, New Jersey) and was placed at the air–liquid interface for 14 days in FAD medium supplemented with SCF (10 ng/mL), EDN3 (100 nM), FSK (20 µM), αMSH trifluoroacetate 0.1 µM, and 50 μg/mL ascorbic acid. For the in vivo experiments, skin equivalents were grafted onto the back of 12-week-old male Swiss mice (Charles River Laboratories, Wilmington, MA, USA), as previously described [39]. Implants were harvested 2 weeks after grafting, and the tissue specimens were fixed in 10% buffered formalin (Sigma-Aldrich) for paraffin embedding. The grafting protocol was approved and authorized by CIEMAT.

Histology. Paraffin sections were stained with hematoxylin and eosin (H&E) using standard protocols.

Fontana Masson staining. Paraffin-embedded sections were deparaffined using Ventana BenchMark XT according to the manufacturing datasheets and stained using a Melanin Staining Kit (Abcam; ab150669, Cambridge, UK) according to the manufacturer’s protocol.

Immunohistochemistry. Experiments were performed using Ventana BenchMark XT according to the manufacturing datasheets. Briefly, sections were stained using 1/100 mouse anti-Involucrine (Sigma-Aldrich, St. Louis, MO, USA. I9018) and 1/100 rabbit anti-TYRP1 (Lsbio; LSB4011), 1/100 mouse anti-Tyrosinase (Abcam; ab738, Cambridge, UK), and 1/500 rabbit anti-KRT14 (Biolegend, San Diego, CA, USA; PRB-155-P) antibodies for 1 h at RT. After the wash cycles, secondary antibody 1:1000 goat anti-mouse 555 and goat anti-mouse 488 (ThermoFisher, Waltham, MA, USA) were applied together with DAPI 1:10,000 (ThermoFisher, Waltham, MA, USA) for 1 h at RT. After washing, slides were mounted in Fluoromount-G™ Mounting Medium (ThermoFisher, Waltham, MA, USA). Images were captured using a Zeiss epifluorescence illumination microscope.

Melanin quantification. Mel-CSP was cultured at 1 × 10^5^ cells per well in a 6-well plate for 48 h. After centrifugation, the cells pellets were dissolved in 1N NaOH (Sigma-Aldrich) for 1 h at 65 °C. After centrifugation at 12,000× *g* for 10 min, the supernatants were transferred to 96-well plates with a standard curve (0–50 µg/mL) prepared from synthetic melanin diluted in 1N NaOH. The melanin content was measured by absorbance at 405 nm on the microplate reader Clariostar (BMGLabtech, Ortenberg, Germany) with its softwares (Clariostar^®^ v5.70 R3 and MARS® v4.01 R2). Melanin contents were analyzed according to a linear regression line obtained with a graduated concentration of synthetic melanin.

Electron Microscopy (EM). Cells were seeded on a culture plate and fixed with 2% (*v*/*v*) glutaraldehyde (Sigma-Aldrich) in 0.15 M Sorensen buffer (Sigma-Aldrich) for 24 h. Briefly, cells were rinsed with cacodylate buffer (Sigma-Aldrich), post-fixed in osmium tetroxide (Sigma-Aldrich), dehydrated in increased concentrations of ethanol (Sigma-Aldrich), and embedded in EPON resin (TAAB, Aldermaston, Berkshire, Omaha, NE, USA) while on coverslips. Ultrathin sections were contrasted with uranyl acetate (Sigma-Aldrich) and citrate (Sigma-Aldrich) then observed under an electron microscope (CM120; FEI, Lausanne, Switzerland). Micrographs were taken with a KeenView digital camera (Soft Imaging System, Denver, CO, USA) and analySIS^®^ software (version 3.1, Soft Imaging System).

AmpliSeq Cancer Hotspot Panel v2. The cDNA library was constructed using Ion AmpliSeq Cancer Hotspot Panel v2 and Ion AmpliSeq Library kit v2 and barcoded using an Ion Xpress Barcode Adapter (ThermoFisher, Waltham, MA, USA). The samples were quantified using an Agilent High-Sensitivity DNA kit and sequenced on an Ion Proton platform using an Ion PI Hi-Q sequencing 200 kit chemistry. The sequencing results were aligned, and variant caller analysis was generated using Ion Torrent suite software and annotated on the COSMIC database or FATHMM database.

AmpliSeq sequencing. The cDNA library was constructed using the Ion AmpliSeq Transcriptome Human Gene Expression kit and barcoded using an Ion Xpress Barcode Adapter (ThermoFisher). The samples were quantified using an Agilent High-Sensitivity DNA kit and sequenced on an Ion Proton platform using an Ion PI Hi-Q sequencing 200 chemistry kit. The sequencing results were aligned on an hg19 and analyzed with Partekflow (v6 Partek Inc., Chesterfield, MO, USA) and Partek Genomic Suite.

Kinetic Analysis. Quality control was performed using FastQC (v0.11.2) [40]. FastQC is a quality control tool for high-throughput sequence data).

Sequencing reads were trimmed with PRINSEQ (v0.20.4) (--trim-right 20) and filtered by average quality score (--trim-qual 20). Reads were mapped to the *H. sapiens* reference genome (hg19) and GRCh37.75 EnsEMBL reference annotation by RNA-STAR (v2.4.1d) [41]. Reads below a mapping score of 10 or multi-mapped were filtered using SAMtools (v0.1.19) [42]. The gene expression level in each sample was calculated with HTSeq-count (v0.7.2, Python 2.7) [43], and differential expression between states of differentiation was computed with DESeq2 (v1.10.1 using R v3.2.4) [39]. We considered genes as differentially expressed when their adjusted *p*-values are lower than 0.05 and their |log2FoldChange| values are larger 0.4. The kinetic study was performed with the ImpulseDE package (R v3.4.3), https://github.com/YosefLab/ImpulseDE, accessed on 10 January 2018, in single time course mode and impulse DE function [44]. We used the normalized z-score standardized expressions as the input, in samples of the 618 significantly differentially expressed genes in common with every comparison (day 0 versus day 30, day 7 versus day 30, day 14 versus day 30 and day 21 versus day 30).

Soft agar assay for colony formation. A base 0.5% agar (Sigma-Aldrich) was prepared under a top 0.3% agar containing cells seeded at 25,000/cm^2^. Plates were incubated at 37 °C in a humidified incubator for 30 days. Cells were fed 1–2 times per week with cell culture media (DMEM + glutamax + 10% FBS, all from ThermoFisher. At day 30, plates were stained with 0.5 mL of 0.005% Crystal Violet (Sigma-Aldrich) for 1.5 h. Colonies were observed and counted under a dissecting microscope.

Karyotype (G-banding). A total of 10^6^ cells were blocked in metaphase by adding colchicine (Eurobio, FR) at a final concentration of 1 mg/L for 90 min and then washed twice with PBS. They were detached from the culture dish using an incubation of 5 min with 0.05% trypsin/EDTA, and the resulting cell suspension was transferred into a 15 mL falcon tube and centrifuged. The supernatant was discarded, and the pellet was suspended in 8 mL of hypotonic solution of KCl (5.6 mg/mL) (Sigma-Aldrich) for 25 min at 37 °C and fixed with Carnoy solution (3v methanol/1v acetic acid, all from Sigma-Aldrich. Drops of cell suspensions were spread on several Superfrost histological slides and allowed to dry at room temperature overnight. For G banding, slides were placed directly in a trypsin solution 1× for 25 s, rinsed quickly in two baths of PBS, and then stained with Giemsa (Sigma-Aldrich) for one minute and rinsed under running water. We used our Metasystem platform to identify the metaphases via the Metafer 4 program (version v3.11.8WK). Metaphases were photographed with an AxioImager Zeiss Z2 microscope combined with a camera cool cube and 10× and 63× objectives. For G banding, 50 metaphases were analyzed with Ikaros software (version v5.7.8 WK) and chromosomes were classified into 6 metaphases. For mFISH, hybridization was performed according to the supplier’s protocols (MetaSystems, Altlussheim Germany) and slides were incubated overnight with 24× Cyte Human Multicolor FISH Probes. Images were captured with MetaSystems platforms and were analyzed with Isis software (version v5.7.8 WK, MetaSystems).

## Figures and Tables

**Table 1:** Description of gene clusters. Relative to Figure 4.

Clusters Description													
Cluster 1		Cluster 2		Cluster 3	Cluster 4	Cluster 5					Cluster 6	Cluster 7	Cluster 8
*LAS1L*	*CLDN3*	*LIG3*	*FOXB1*	*TNFRSF9*	*HSPA5*	*ATP10A*	*CEBPD*	*HSBP1L1*	*GAS2*	*NPAS2*	HIST1H4B	HMGA1	TPX2
*CRLF1*	*MYO5B*	*TACC3*	*AFF1*	*C6orf186*	*CD81*	*HSPB6*	*LGALS3BP*	*SERPINF1*	*ENDOD1*	*PDGFD*	HNRNPU	HIST1H2BD	CBX5
*CD9*	*WDR81*	*FLT4*	*HES3*	*OPRK1*	*MYL12B*	*TBXA2R*	*SLC16A6*	*TBC1D14*	*ZC3H12C*	*JUNB*	HIST1H2BI	HIST1H3I	SFRP1
*KAL1*	*PCSK9*	*POLD1*	*CBX2*	*LTBP4*	*CD63*	*GAS7*	*PMP22*	*BCAN*	*OXSM*	*PTEN*	HIST1H1C	HIST1H2AH	FBL
*NLRP2*	*KRT8*	*SUGP2*	*MSL2*	*GALNTL1*	*MGST3*	*CYTH3*	*ST3GAL4*	*TMCC2*	*CCDC3*	*ISG20*	HIST1H2BM	SMCHD1	CENPF
*CDH3*	*XKR6*	*MCM10*	*UNC119B*	*CHRDL1*	*MMP14*	*NFIX*	*ELMOD1*	*TRPM1*	*ACSL1*	*RCAN2*	HIST1H2BE		SNRPF
*PRKCZ*	*ZIK1*	*GLI2*	*LMNB2*	*COTL1*	*COL1A2*	*LRRC23*	*CLEC2B*	*SORT1*	*TXNDC11*	*IL16*			G3BP1
*SRRT*	*BNC2*	*TFAP4*	*ZNF678*	*FZD3*	*PPIB*	*MVP*	*LTBR*	*CABLES1*	*DAB2*	*EHBP1L1*			RCOR2
*KDM2B*	*ZNF483*	*KIF4A*	*GINS3*	*LGI1*	*CTNNB1*	*SNAI2*	*GPR133*	*EMP1*	*C9orf150*	*CSPG4*			UBE2C
*CYP26A1*	*PSMD2*	*SRPK1*	*FIGN*	*DBX1*	*SILV*	*GPR124*	*COL12A1*	*RAB33A*	*FAM134B*	*YIF1A*			HIST1H2AC
*ITPR3*	*GSG2*	*DSP*	*IQGAP3*	*ZBTB16*		*RUNX3*	*NCOA7*	*P2RX4*	*C10orf90*	*A2M*			HIST1H2BG
*CECR2*	*GLDC*	*NUP93*	*HIST1H1B*	*PARD3B*		*ABCC2*	*SMOC2*	*CD164*	*ADAMTS1*	*KLHL38*			HIST1H4D
*SALL4*	*ARID3B*	*DNMBP*	*KIF18B*	*HOXB1*		*ELN*	*LOX*	*AGT*	*FGD5*	*NUPR1*			HIST1H2BH
*HAGHL*	*FGD6*	*MAP2K6*	*LIN28B*	*NPPB*		*HEXB*	*PDE4D*	*FAM129A*	*ACSS1*	*RTTN*			HIST1H3A
*CTSH*	*ERCC6L*	*NCAPG*	*TCF4*	*WFIKKN1*		*CCDC90A*	*PDGFRB*	*SERPINE2*	*CA10*	*FIBIN*			HIST1H2AL
*ESRP1*	*SEMA4D*	*PTGES3*	*WNK3*	*DNAJB1*		*HERPUD1*	*CPEB4*	*CYP27A1*	*MARVELD1*	*DPP7*			
*GSDMD*	*ARID2*	*LMNB1*	*HIST1H4I*	*WASF3*		*BRP44L*	*ROPN1B*	*ARMC9*	*GOLGA7B*	*LRRN4CL*			
*LIG1*	*HIST1H3E*	*UXS1*	*HIST1H4L*	*NAV1*		*IDH3G*	*PLSCR4*	*LMO7*	*CD109*	*GBA*			
*SIPA1L3*	*SIPA1L1*	*ARID3A*	*SMOC1*	*SOX3*		*RAB27A*	*STAM2*	*SETDB2*	*FBXO32*	*C7orf41*			
*UNC5B*	*ZNF770*	*EPHA4*	*LGR4*	*LGR5*		*WIPI1*	*KCNJ13*	*GPNMB*	*FZD1*	*MAB21L1*			
*SLC9A3R1*	*WDHD1*	*C1orf109*	*CCDC85C*	*ATBF1*		*ST6GALNAC2*	*MLPH*	*NDUFB5*	*C9orf91*	*GREM2*			
*PVRL1*	*DAXX*	*ARID1A*	*SMN2*	*BOC*		*SEL1L*	*PLCL1*	*ABI1*	*TNFRSF14*	*SLC9A9*			
*SLC38A1*	*EIF5AL1*	*KDM3B*	*NEURL1B*	*SFRP2*		*CYBRD1*	*SLC1A4*	*STXBP1*	*TRIM63*	*AP1S2*			
*NUP155*		*LYPLA1*	*HIST1H2AM*	*SCUBE3*		*CDH19*	*TRAK2*	*NOV*	*CYB5R1*	*NTM*			
*GALNT3*		*NCAPH*	*KIFC1*	*CDKN2B*		*SLC6A15*	*SCAMP3*	*TFAP2A*	*SPON2*	*TMEM119*			
*MSH6*		*ZWINT*		*CRB2*		*PDE8A*	*GBP1*	*IRF4*	*S100B*	*SOCS3*			
*HDAC1*		*CIT*		*AP000654.2*		*PTGS2*	*RAB32*	*CPEB2*	*LMNA*	*TMEM173*			
*OLFML3*		*PPAT*		*TMEM132D*		*TYR*	*KLF9*	*SULF1*	*C19orf28*	*NDUFA4L2*			
*TTF2*		*SMO*		*WNT7A*		*GPR137B*	*ITGB1BP1*	*THBS1*	*JOSD2*	*SHC4*			
*RCAN3*		*CNN1*		*DPYSL5*		*FAP*	*NR4A3*	*IFI44*	*GRASP*	*KCNQ5*			
*PLXDC2*		*NUP210*		*KLHDC8A*		*CEACAM1*	*RHOQ*	*ANXA7*	*NFIA*	*IRS2*			
*PLS1*		*PIK3C2B*		*PTX3*		*STX7*	*IFIT3*	*ARHGAP24*	*TM2D1*	*THBS2*			
*HNRNPA2B1*		*CDCA8*		*MAMDC2*		*DCT*	*MLANA*	*CPNE8*	*LHX8*	*C6orf1*			
*STIL*		*C9orf100*		*CENPV*		*EPDR1*	*C5orf32*	*C1RL*	*DDR2*	*MITF*			
*PAIP2B*		*HIST1H2AB*		*HOXB9*		*PPP1R15A*	*TGFBI*	*PHLDA1*	*KCNF1*	*CYP26C1*			
*RRBP1*		*MDC1*		*SNCG*		*COQ9*	*GLT8D2*	*FBLN5*	*GPR155*	*TRPV2*			
*EGLN3*		*MAP2K5*		*NRIP3*		*P2RX7*	*SOCS2*	*BCL2A1*	*IGFBP7*	*THSD4*			
*MLLT4*		*SHROOM3*		*IRX5*		*SLC17A6*	*SORBS3*	*DNAJA4*	*IFI16*	*FAM69C*			
*ASS1*		*LRIG3*		*IRX3*		*GADD45B*	*TBX2*	*ABHD2*	*ARHGEF3*	*DNER*			
*C19orf66*		*STON2*		*NRIP1*		*PALM*	*PDZRN3*	*TGFB1I1*	*EMCN*	*CHM*			
*NOL11*		*C15orf42*		*EXT1*		*MMP11*	*GJA3*	*SAT2*	*SLC45A2*	*SLC24A5*			
*LIN28A*		*ZNF710*		*HIST2H2BE*		*SLC7A4*	*UBL3*	*TTYH2*	*EDIL3*	*PARVB*			
*CC2D1A*		*MED9*		*COL25A1*		*LGALS1*	*LRRC39*	*NFIC*	*SHH*	*LRRK2*			
*HRSP12*		*POU2F1*		*FAT4*		*SOX10*	*INHBA*	*TMEM50B*	*BRI3*	*APOD*			
*NASP*		*SOX13*		*VEPH1*		*TIMP3*	*SRGN*	*EMP3*	*CTSB*	*TDRD7*			
*SYT6*		*SKP2*		*HOXC6*		*TSPO*	*BICC1*	*ECM1*	*NOS3*	*MME*			
*DSC2*		*LIX1*		*ELOVL2*		*ATP6V1D*	*BHLHE41*	*CTSK*	*CRYL1*	*CASP4*			
*HEY2*		*ADAMTS19*		*DLL1*		*CINP*	*ZC3H13*	*DUSP10*	*C10orf10*	*ANXA4*			
*USP44*		*ZMYM4*		*POU3F3*		*CTSZ*	*RAB38*	*LYST*	*MFAP4*	*METTL9*			
*LECT1*		*NR6A1*				*BIRC7*	*F13A1*	*ARL8A*	*NNMT*	*ANXA6*			
*SORL1*		*QTRTD1*				*TRIB3*	*SERPINB6*	*MANF*	*SLFN5*	*SLC6A17*			
*FRAS1*		*UTRN*				*PLP2*	*SBDS*	*EPHA5*	*STAT6*	*LRP10*			
*PTPRF*		*SKA1*				*RENBP*	*SIX1*	*SNCA*	*AKTIP*	*UAP1L1*			
*MCL1*		*SFRS15*				*ASB9*	*MASP1*	*DDIT4L*	*SLC3A2*	*DACT3*			
*CDCA5*		*PPP1R9A*				*ACP5*	*ZFP36*	*OSMR*	*LTBP3*	*GSTK1*			
*LPCAT1*		*IGF2BP1*				*PLA2G15*	*VGF*	*CXCL14*	*LGI3*	*MBP*			
*SLC16A1*		*DBF4B*				*PGCP*	*DNAJB9*	*TBC1D7*	*SLC16A4*	*CHSY3*			
*DUSP2*		*C1orf187*				*NIPAL2*	*LRRC17*	*LRRTM2*	*NSG1*	*MMP17*			
*C21orf45*		*C1orf106*				*RAB2A*	*ISLR*	*DAAM2*	*C6orf145*	*C1orf85*			
*ACTC1*		*PPM1L*				*ASAH1*	*RNASE1*	*ARHGAP18*	*ATF5*	*EGFL6*			
*FGFR4*		*MAD2L1*				*CAV2*	*GDF15*	*C6orf192*	*IL1RAPL1*	*SHISA4*			
*PDPN*		*USP49*				*PCOLCE*	*NDFIP1*	*SDK1*	*SLC33A1*	*TGM2*			
*PKDCC*		*PLEKHA7*				*AHR*	*UBE2D2*	*TACC1*	*IL8*	*CARD16*			
*PQLC3*		*HIC2*				*AEBP1*	*TMEM204*	*EBAG9*	*HMP19*	*CFI*			
*ZNF589*		*IRX2*				*SFXN3*	*LGALS3*	*NFIB*	*CRTAP*				
*CAMKV*		*PPID*				*DNAJC12*	*MATN3*	*GSN*	*SEMA3E*				

**Table 2:** Primers used in qPCR analysis.

Gene Name	Forward	Reverse
*18S*	GAGGATGAGGTGGAACGTGT	TCTTCAGTCGCTCCAGGTCT
*S0X10*	AGCCCAGGTGAAGACAGAGA	ATAGGGTCCTGAGGGCTG AT
*MITF*	GCTCACAGCGTGTATTTTTCC	TCTCTTTGGCCAGTGCTCTT
*TYRP1*	AGCAGTAGTTGGCGCTTTGT	TCAGTGAGGAGAGGCTGGTT
*TYR*	TTGTACTGCCTGCTGTGGAG	CAGGAACCTCTGCCTGAAAG
*PMEL17*	TGGACAGAAGCCCAGAGACT	GCAATACCTTTTGGCTTCCA
*DCT*	GGTTCCTTTCTTCCCTCCAG	AACCAAAGCCACCAGTGTTC
*RAB27A*	GGGCAGGAGAGGTTTCGTAG	TGCATGCATCTGTAGCTGGC

## Data Availability

The raw and analyzed datasets generated during the study are available for research purposes from the corresponding authors. Transcriptomic datasets related to this article can be found at https://www.ncbi.nlm.nih.gov/geo/query/acc.cgi?acc=GSE222639 hosted by the Gene Expression Omnibus database (accession number GSE222639).

## Supplementary Materials

The following supporting information can be downloaded at: https://www.mdpi.com/article/10.3390/ijms24076398/s1.
