# Supplementary material for: Generating Functional and Highly Proliferative Melanocytes Derived from Human Pluripotent Stem Cells: A Promising Tool for Biotherapeutic Approaches to Treat Skin Pigmentation Disorders
Source: Int J Mol Sci. 2023 Mar 29;24(7):6398. doi: 10.3390/ijms24076398 (PMC10094141; doi:10.3390/ijms24076398)
Supplement: Supplementary file 1 [file ijms-24-06398-s001.zip › ijms-2273757-supplementary.pdf]

i		
GO BP	fold enrichment	Pvalue
melanin metabolic process (GO:0006582)	8.61	1.75×10^{-2}
melanocyte différentiation (GO:0030318)	6.58	4.54×10^{-2}
Développemental pigmentation (GO:0048066)	6.06	4.21×10^{-5}
pigment cell différentiation (GO:0050931)	6.05	2.90×10^{-5}
pigmentation (GO:0043473)	4.68	1.45×10^{-8}
cellular pigmentation (GO:0033059)	4.57	3.59×10^{-3}

ii		
GO BP	fold Enrichment	P-value
negative regulation of viral process (GO:0048525)	7.53	3.30×10^{-3}
negative regulation of viral life cycle (GO:1903901)	7.34	4.62×10^{-2}
small molecule metabolic process (GO:0044281)	1.98	2.67×10^{-2}
cellular metabolic process (GO:0044237)	1.35	2.98×10^{-2}

iii		
GO BP	fold Enrichment	P-value
establishment of protein localization (GO:0045184)	1.78	1.03×10^{-2}
intracellular transport (GO:0046907)	1.78	1.23×10^{-2}
protein transport (GO:0015031)	1.78	2.22×10^{-2}
peptide transport (GO:0015833)	1.75	3.90×10^{-2}

Figure S1: Gene ontology biological process of mature melanocytes. Related to figure 1.
Gene ontology biological process (GO BP) terms from indicated regions (i, ii and iii) of Venn diagram described in figure 1(f).

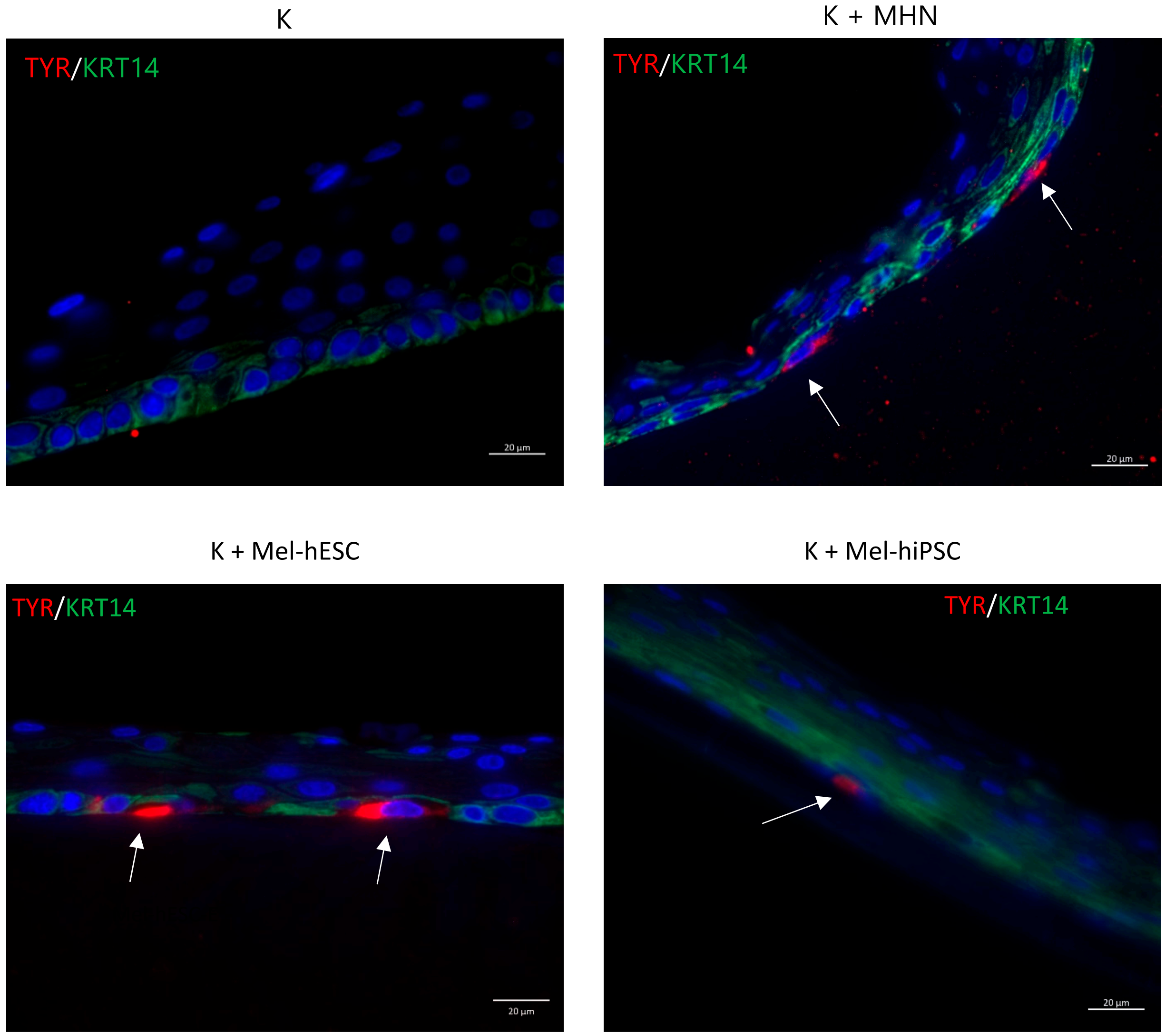

Figure S2: Localization of Mel-hPSC and keratinocytes in epidermal basal layer. Related to figure 2.
Immunofluorescence staining of Keratin 14 (KRT14) and Tyrosinase (TYR) on sections of the *in-vitro* reconstructed epidermis containing HEM, Mel-hESC or Mel-hiPSC (Scale bar: 20μm). White arrows indicate TYR staining in MHN, Mel-hESC and Mel-hiPSC

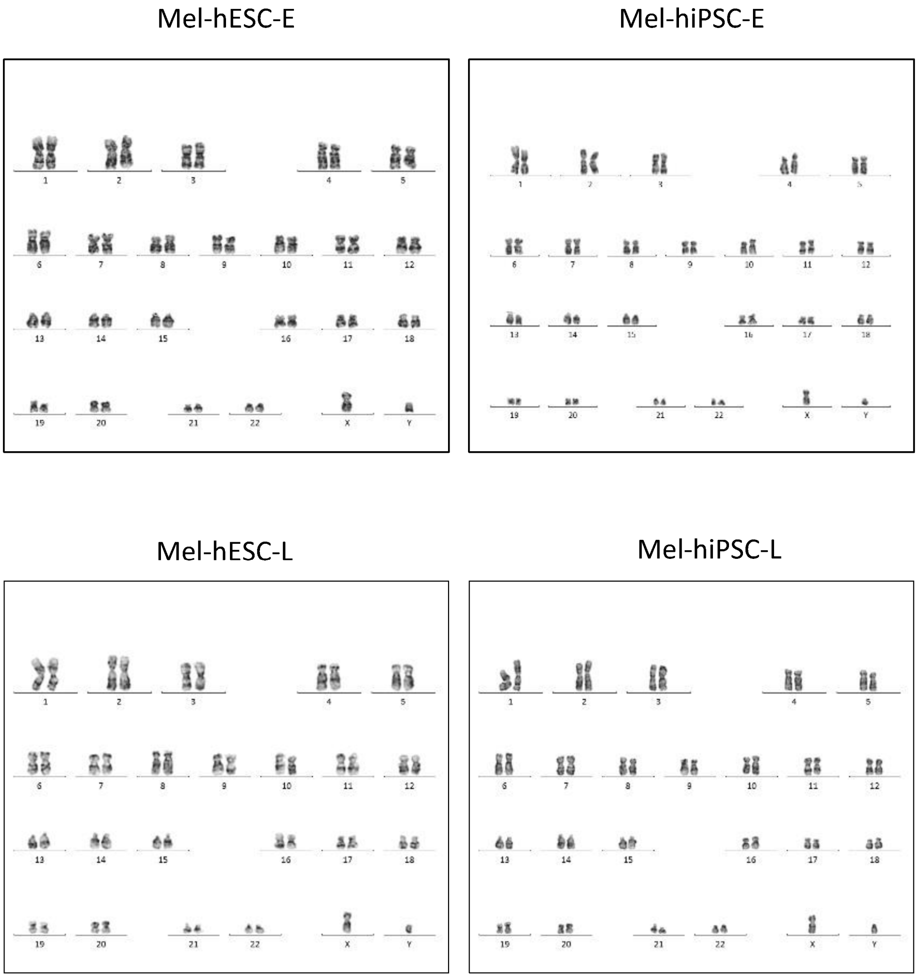

Figure S3: Karyotype analysis by G-banding Related to figure 3
G-banding analysis in Mel-hESC and Mel-hiPSC at early and late passages.

Cluster #1 (C1)

GO category	GO terms	pValue
Biological process	tissue development (GO:0009888)	$4{,}89 \times 10^{-7}$
	epithelium development (GO:0060429)	$2{,}64 \times 10^{-6}$
	tissue morphogenesis (GO:0048729)	$2{,}30 \times 10^{-5}$
	cellular component morphogenesis (GO:0032989)	$2{,}99 \times 10^{-5}$
	embryo development (GO:0009790)	$3{,}97 \times 10^{-5}$
	regulation of neuron death (GO:1901214)	$4{,}52 \times 10^{-5}$
	tube development (GO:0035295)	$4{,}56 \times 10^{-5}$
	morphogenesis of an epithelium (GO:0002009)	$7{,}59 \times 10^{-5}$
	embryonic organ development (GO:0048568)	$8{,}15 \times 10^{-5}$
	sensory organ development (GO:0007423)	$8{,}81 \times 10^{-5}$
Molecular function	NS.	NS.
Cellular component	NS.	NS.

Cluster #2 (C2)

GO category	GO terms	pValue
Biological process	mitotic cell cycle process (GO:1903047)	$9{,}00 \times 10^{-10}$
	mitotic cell cycle (GO:0000278)	$1{,}22 \times 10^{-9}$
	mitotic sister chromatid segregation (GO:0000070)	$3{,}69 \times 10^{-8}$
	mitotic nuclear division (GO:0140014)	$2{,}70 \times 10^{-7}$
	sister chromatid segregation (GO:0000819)	$2{,}70 \times 10^{-7}$
	cell division (GO:0051301)	$2{,}89 \times 10^{-7}$
	chromosome segregation (GO:0007059)	$3{,}64 \times 10^{-7}$
	nuclear chromosome segregation (GO:0098813)	$6{,}28 \times 10^{-6}$
	nuclear division (GO:0000280)	$3{,}62 \times 10^{-5}$
	organelle fission (GO:0048285)	$7{,}03 \times 10^{-5}$
Molecular function	NS.	NS.
Cellular component	NS.	NS.

Cluster #3 (C3)

GO category	GO terms	pValue
Biological process	canonical Wnt signaling pathway (GO:0060070)	$3{,}22 \times 10^{-5}$
	negative regulation of neurogenesis (GO:0050768)	$7{,}84 \times 10^{-5}$
	nervous system development (GO:0007399)	$1{,}15 \times 10^{-4}$
	Wnt signaling pathway (GO:0016055)	$1{,}52 \times 10^{-4}$
	negative regulation of embryonic development (GO:0045992)	$3{,}19 \times 10^{-4}$
	generation of neurons (GO:0048699)	$2{,}96 \times 10^{-4}$
	establishment or maintenance of apical/basal cell polarity (GO:0035088)	$9{,}79 \times 10^{-4}$
	regulation of TGF -beta receptor signaling pathway (GO:0017015)	$9{,}94 \times 10^{-4}$
Molecular function	NS.	NS.
Cellular component	NS.	NS.

Cluster #4 (C4)

GO category	GO terms	pValue
Cellular component	melanosome (GO:0042470)	$1{,}32 \times 10^{-6}$
	pigment granule (GO:0048770)	$1{,}32 \times 10^{-6}$
	focal adhesion (GO:0005925)	$9{,}04 \times 10^{-4}$
	cell-substrate adherens junction (GO:0005924)	$9{,}37 \times 10^{-4}$
	cell-substrate junction (GO:0030055)	$9{,}83 \times 10^{-4}$
	vesicle (GO:0031982)	$2{,}58 \times 10^{-3}$
	adherens junction (GO:0005912)	$3{,}68 \times 10^{-3}$
	anchoring junction (GO:0070161)	$4{,}25 \times 10^{-3}$
	extracellular region (GO:0005576)	$8{,}09 \times 10^{-3}$
Molecular function	N S.	NS.
Biological process	NS.	NS.

Cluster #5 (C5)

GO category	GO terms	pValue
Biological process	negative regulation of cell adhesion (GO:0007162)	$2{,}10 \times 10^{-6}$
	developmental pigmentation (GO:0048066)	$4{,}07 \times 10^{-4}$
	pigmentation (GO:0043473)	$6{,}27 \times 10^{-4}$
	platelet degranulation (GO:0002576)	$3{,}64 \times 10^{-3}$
	extracellular matrix organization (GO:0030198)	$9{,}39 \times 10^{-3}$
Cellular component	extracellular matrix (GO:0031012)	$4{,}97 \times 10^{-18}$
	collagen-containing extracellular matrix (GO:0062023)	$7{,}54 \times 10^{-17}$
	melanosome (GO:0042470)	$1{,}48 \times 10^{-7}$
	pigment granule (GO:0048770)	$1{,}48 \times 10^{-7}$
	lysosome (GO:0005764)	$1{,}10 \times 10^{-6}$
	melanosome membrane (GO:0033162)	$3{,}28 \times 10^{-5}$
	chitosome (GO:0045009)	$3{,}28 \times 10^{-5}$
	pigment granule membrane (GO:0090741)	$3{,}28 \times 10^{-5}$
	platelet alpha granule (GO:0031091)	$9{,}69 \times 10^{-4}$
Molecular function	extracellular matrix structural constituent (GO:0005201)	$6{,}01 \times 10^{-5}$
	glycosaminoglycan binding (GO:0005539)	$4{,}27 \times 10^{-3}$
	protein homodimerization activity (GO:0042803)	$4{,}30 \times 10^{-3}$

[illegible]

Figure S4: Clusters enrichment terms during differentiation process. Related to figure 4.
Enrichment terms from Gene ontology analysis, biological process (GO BP), molecular function (MF) and cellular component (CC) terms from the list of each 5 clusters. pValue <0,01 and number max =10 terms.
